# Dimensional Accuracy in 3D Printed Medical Models: A Follow-Up Study on SLA and SLS Technology

**DOI:** 10.3390/jcm13195848

**Published:** 2024-09-30

**Authors:** Bilal Msallem, Joel J. Vavrina, Michel Beyer, Florian S. Halbeisen, Günter Lauer, Adrian Dragu, Florian M. Thieringer

**Affiliations:** 1 UniversityCenter for Orthopedics, Trauma and Plastic Surgery, Faculty of Medicine and University Hospital Carl Gustav Carus, TUD Dresden University of Technology, DE-01307 Dresden, Germany; adrian.dragu@ukdd.de; 2 Medical Additive Manufacturing Research Group, Department of Biomedical Engineering, University of Basel, CH-4123 Allschwil, Switzerland; joel.vavrina@unibas.ch (J.J.V.); michel.beyer@usb.ch (M.B.); florian.thieringer@usb.ch (F.M.T.); 3 Clinic of Oral and Cranio-Maxillofacial Surgery, University Hospital Basel, CH-4031 Basel, Switzerland; 4 Basel Institute for Clinical Epidemiology and Biostatistics, Department of Clinical Research, University Hospital Basel, University of Basel, CH-4031 Basel, Switzerland; 5 Department of Oral and Maxillofacial Surgery, Faculty of Medicine and University Hospital Carl Gustav Carus, TUD Dresden University of Technology, DE-01307 Dresden, Germany; guenter.lauer@ukdd.de

**Keywords:** 3D printing, computer-aided design, dimensional measurement accuracy, precision medicine, stereolithography

## Abstract

**Background:** With the rise of new 3D printers, assessing accuracy is crucial for obtaining the best results in patient care. Previous studies have shown that the highest accuracy is achieved with SLS printing technology; however, SLA printing technology has made significant improvements in recent years. **Methods:** In this study, a realistic anatomical model of a mandible and skull, a cutting guide for mandibular osteotomy, and a splint for orthognathic surgery were replicated five times each using two different 3D printing technologies: SLA and SLS. **Results:** The SLA group had a median trueness RMS value of 0.148 mm and a precision RMS value of 0.117 mm. The SLS group had a median trueness RMS value of 0.144 mm and a precision RMS value of 0.096 mm. There was no statistically significant difference in RMS values between SLS and SLA technologies regarding trueness. Regarding precision, however, the RMS values for SLS technology were significantly lower in the splint and cutting guide applications than those printed with SLA technology. **Conclusions:** Both 3D printing technologies produce modern models and applications with equally high dimensional accuracy. Considering current cost pressures experienced by hospitals, the lower-cost SLA 3D printer is a reliable choice for point-of-care 3D printing.

## 1. Introduction

Advancements in computer-aided design (CAD) and computer-aided manufacturing (CAM) have considerably enhanced the capabilities of additive manufacturing (AM) [1,2]. AM employs a process of material deposition based on virtual planning data to fabricate physical objects, typically in a layer-by-layer fashion [3]. AM is widely applied across numerous sectors and is increasingly important in the healthcare industry [4,5,6]. According to international ISO standards, AM can be categorised into seven distinct classes [3], one of which includes VAT-photopolymerisation technology. This technology involves the selective curing of a liquid photopolymer via light-activated polymerisation; this process constructs the object layer by layer [3]. Stereolithography (SLA) is particularly noteworthy within this category and is the most commonly employed technology across all AM categories [7,8]. Another prominent category is powder bed fusion technology, which utilises a high-power laser to sinter powdered materials incrementally, leading to partial fusion of the material [3,5]. Selective laser sintering (SLS) is frequently utilised alongside SLA in healthcare applications [1,9]. Both technologies have different advantages and disadvantages, which enable their potential use in various medical fields [5]. For example, SLS technology enables higher design freedom because no support structures are required (unlike SLA technology). A disadvantage of SLS technology is the rough surface finish compared to the smooth surface finish possible with SLA technology [10].

These technologies enable the production of diverse anatomical models, implants, and other healthcare applications, which are becoming fundamental components of the medical industry [5,6]. For instance, customised anatomical models are instrumental in planning and training for surgical procedures [11,12], which in turn reduce operating time and complication rates [8,13,14]. These models enhance practitioners’ understanding of human anatomy [15,16] and aid in selecting appropriate surgical applications and procedures [17,18]. Individual 3D printed models provide superior educational value over digital images not only by highlighting specific anatomical features but also by facilitating visual and tactile learning processes, thereby enhancing anatomical comprehension and accelerating the learning process [15,19]. Additionally, 3D printed models are more durable and can be preserved for longer than organic tissues without significant degradation [5,20]. Finally, it is possible to manufacture patient-specific anatomical prostheses, stents and surgical tools, such as instruments and cutting guides for mandibular osteotomy or orthognathic surgery splints [5,6,17,21]. The precision of these tools and models is of critical importance within healthcare. The accuracy specifications of 3D printer manufacturers are determined primarily using isosymmetrically shaped test bodies [22,23]. However, determining accuracy in asymmetrical test bodies (such as anatomical models) is more challenging.

Despite advancements, a systematic review spanning 158 studies from 2005 to 2015 highlighted that the accuracy of 3D printed anatomical models, surgical guides, and implants in maxillofacial and orthopaedic surgeries was unsatisfactory in 34 studies [24]. Among 3D printed skull models, measurements showed deformations and missing parts in some prints [25,26]; such inaccuracies can lead to improper treatments and may pose risks to patients. This shows that identical 3D printing jobs do not always result in similar outcomes. Model size, height, and layering contribute to a ‘stair-stepping error’, which worsens with increased layer count and thickness [22,27,28]. Studies indicate that the positioning and alignment of models during printing significantly affect the accuracy and structural stability of the final product, influencing factors like printing angle as well as layer direction and distance to the print centre [29]. In addition to process-inherent deviations [29,30,31,32,33], discrepancies between 3D printed models and their anatomical counterparts can arise from several sources, including the low resolution of radiological imaging, segmentation inaccuracies, or errors introduced during the conversion of DICOM files to STL files and subsequent STL manipulation [34,35]. Such deviations underline the challenges of digitalisation, such as manual registration or digital measurement inaccuracies [26,36].

This study aims to build on previous research by Msallem et al., which compared several established and widely used 3D printing technologies [9]. This study addresses a current and pressing issue, as evidenced by the high demand for previous research. Because the technologies analysed in the previous study have since evolved, this study again examines the 3D printing technologies with the highest and lowest accuracies (in terms of precision and trueness). The printing accuracies of two printing technologies (an SLS 3D printer (Fuse 1) and an SLA 3D printer (Form 3B+)) were compared. Four different applications for medical use were printed, all of which differ in size and shape. Some have recesses or openings, while others have smooth or rough surfaces. Deviation patterns concerning the material used and the appropriate application are analysed.

Finally, conclusions are reached for each printing technology, and sources of error are discussed to aid practitioners in selecting the most appropriate technology for their clinical needs.

## 2. Material and Methods

In this study, an anatomical model of the mandible and skull, a cutting guide for mandibular resection, and a splint for orthognathic surgery were replicated five times each using two different printing technologies (*n* = 40), namely SLA and SLS. The replicas were measured and compared with the reference data (*n* = 40) and with each other (*n* = 80). By such means, the accuracy of the two 3D printers (in terms of trueness and precision) was determined. A statistical analysis was carried out to assess the closest results between the 3D printed models and the reference data (trueness), as well as the closest results among the different replicas (precision) depending on the printing technology [37]. No ethical approval was required.

### 2.1. Equipment and Material

Data regarding the 3D printer, materials, and layer thickness used in this study are summarised below (Table 1), as are the data concerning the 3D scanner used for digitalisation (Table 2).

The resin used in the SLA printer was always selected to align with the intended purpose. For instance, because the anatomical model is not intended for use during surgical procedures, it was printed with a non-biocompatible material (Standard Grey). By contrast, the splints and cutting guides, which may be used intraoperatively, were printed with a biocompatible material (BioMed Clear). Only the SLA 3D printer has the capability and material range to meet these requirements.

### 2.2. Preparation of the STL File

The digital 3D reference models of the anatomical models (skull and mandible) were created using a computed tomography (CT) dataset of a natural human specimen. By contrast, the 3D reference models of the splint and cutting guide were made using a digital human dataset. To have a representative model, the mandible selected had variances like the loss of tooth 27 (universal numbering system) with an extraction socket and the loss of tooth 20 (universal numbering system) without a visible extraction socket but with bone resorption and several tooth fillings. This digital 3D reference model is the same mandible as that used in the previous comparative study [9].

The standard tessellation language (STL) file (i.e., the virtual 3D model represented as a triangular mesh with the coordinates on a 3D grid) was imported into the 3D slicer software PreForm 3.40 (Formlabs Inc., Somerville, MA, USA), which slices the digital model into cross-sections and generates the print head’s specific path [5]. All models and applications were printed in a vertical orientation, as permitted by the hardware.

### 2.3. Three-Dimensional Printing of the Medical Applications (Replicas)

The 3D printer Fuse 1 (Formlabs Inc., Somerville, MA, USA) produced the models and applications using SLS technology. The material chosen for all models was polyamide powder PA12 (Formlabs Ins., Somerville, MA, USA) with a layer thickness of 110 microns.

The 3D printer Form 3B+ (Formlabs Inc., Somerville, MA, USA) produced the models and applications using SLA technology. Standard Grey resin (Formlabs Inc., Somerville, MA, USA), a grey non-biocompatible material, was chosen for the skulls and mandibular models. BioMed Clear resin (Formlabs Inc., Somerville, MA, USA), a transparent biocompatible resin, was selected for the splints and the cutting guides. The layer thickness for all these models remained constant at 100 microns.

### 2.4. Post-Processing of the Medical Applications (Replicas)

The post-processing of SLS technology involves freeing the components from the powder bed and recycling excess powder via a vibrating screen for further printing processes using the Fuse Sift (Formlabs Inc., Somerville, MA, USA). Finally, the surfaces are smoothed, and any remaining powder residues are removed through sandblasting by the Fuse Blast (Formlabs Inc., Somerville, MA, USA).

The post-processing procedure for SLA technology involves removing uncured resin from the print surface using a Form Wash (Formlabs Inc., Somerville, MA, USA) with a 90% isopropyl alcohol bath for 15 min. The printed object was post-cured for 15 min to improve mechanical strength in the Form Cure (Formlabs Inc., Somerville, MA, USA) with heated, rotating radiation of 405 nm. Finally, the supporting structures were removed using fine-cutting pliers.

### 2.5. Digitisation of the 3D Printed Medical Applications (Replicas)

The 3D printed models were registered and digitised with a Transcan C 3D scanner (Shining 3D Tech. Co., Ltd., Hangzhou, China) using the Solid Edge Shining 3D Edition software 1.4.2.3 (Shining 3D Tech. Co., Ltd., Hangzhou, China). The manufacturer’s specifications included a centre resolution (point distance) of 0.0375 mm on a scan field of 150 mm × 96 mm and a centre resolution (point distance) of 0.075 mm on a scan field of 300 mm × 190 mm (Table 1). The true point distance (calibration deviation) was between 0.05 mm and 0.07 mm after calibration, with a mean deviation of approximately 0.06 mm. The camera resolution was 12 megapixels. The mandibular models, cutting guides, and splints were scanned with a scan field of 150 mm × 96 mm; the skulls were scanned with a scan field of 300 mm × 190 mm. A fully automatic soft light was used for the texture shot. The scanner and the object scanned were protected from ambient stray light. The models were fixed to a rotating turntable until a 360° overview was achieved. This digitisation was performed several times in different positions. To scan the transparent SLA models made with BioMed Clear resin (such as the cutting guides and the splints), a thin layer of a white self-sublimating 3D scanning spray named Reflecon Tarnish 11 (MR Chemie GmbH, Unna, Germany) was applied.

The digitisations were then merged into single digital models, and unwatertight models were created for the splint, cutting guide, and mandibular model. Due to the numerous openings, a watertight model was created for the skulls.

The following specifications were chosen. A non-texture scan was selected, along with high resolution, and the high dynamic range (HDR) was configured to OFF. The turntable was used to control the rotation of the objects using the minimum turntable speed of 1. The align mode was set to features and the automatic global optimisation function was used. The model was meshed with the scanner-integrated software Solid Edge Shining 3D Edition software 1.4.2.3 (Shining 3D Tech. Co., Ltd., Hangzhou, China). The mesh parameters were configured to unwatertight or watertight, depending on the STL. The quality was set to high, the mesh optimisation was set to Filter 2, and the ‘remove small floating parts’ setting was set to 5. The ‘Max triangles’ setting was configured to 20,000,000, and the device was configured to Remove spike. The ‘Maker hole filling’ was configured to OFF, and ‘Recommended parameters’ were set to ON. The generated data were exported in an STL file. Due to the file size, which ranged from a few hundred megabytes (MB) to more than a gigabyte, the scan file was compressed (simplified) to a file size of around 28 MB with around 600,000 polygons.

### 2.6. Accuracy Analysis

Digital measurement was used to avoid the disadvantages of manual measurement and operator variability by having only a few landmarks. Each of the 40 3D printed models and applications (moving entity) was superimposed onto the reference model’s respective application (fixed entity or original data file) using a best-fit alignment method with a 3D analysis programme (3-matic medical v. 15.0, Materialise NV, Leuven, Belgium). All registrations were achieved with an ‘align’ feature by manually placing points for the initial n-point registration. Five registration points were placed for the mandibular models and the splints and four for the skulls and cutting guides. This function first grossly overlaid the two models and applications. Next, to maximise possible superimposition or alignment, a global registration using a semi-automatic algorithm was executed to optimise the distance threshold, the subsample ratio, and the number of iterations. The programme then searched for every surface point in the replica scan file, including the closest point on the surface of the reference model. The distance threshold was set to 0.1 and applied 3 × 100 times until no more repositioning occurred. Subsequently, a part comparison analysis based on a point-based analysis algorithm (closest point) was performed with a maximally tolerated deviation of ±0.5 mm for the cutting guides, splints and mandibular models. The maximal tolerated deviation for the skulls was ±1.0 mm. Root mean square (RMS) values were calculated for each model and application, and the positive as well as negative deviations were determined. Heatmaps were finally created to visualise the areas of aberrance of the original model to the replicas. Analyses for trueness entailed comparing the reference model with all replicas of the two printing technologies (*n* = 40). The precision analysis was performed by comparing all replicas of one printing technology with each other (*n* = 80). In the present study, the reference models and the replicas were computed by calculating the arithmetic square root of the mean squares of a group value between two forms. RMS values were used to quantify systemic error. The larger the RMS values, the greater the deviation from the reference models (and vice versa).

### 2.7. Statistics

A descriptive data analysis summarised mean, standard deviation (SD), median, minimum, maximum, interquartile ranges (IQR), and RMS values for the 3D printed models using both printing technologies. The normality of the RMS values for trueness and precision was assessed using the Shapiro–Wilk test and QQ plots. Because most distributions were non-normal, the Wilcoxon rank sum test was used to compare the RMS values of trueness and precision analysis between printing technologies, considering all models together and each type separately. To address the issue of multiple comparisons, the Holm–Bonferroni correction was applied, which adjusted the significance level for each test to control the family-wise error rate. The level of significance was set to a *p*-value of 0.05. All statistical analyses were performed using the R statistical software (Version 4.2.2, The R Foundation for Statistical Computing, Vienna, Austria).

## 3. Results

### 3.1. Qualitative Accuracy Assessment

#### 3.1.1. Qualitative Accuracy Assessment of SLS Printing Technology

**Figure 1:**
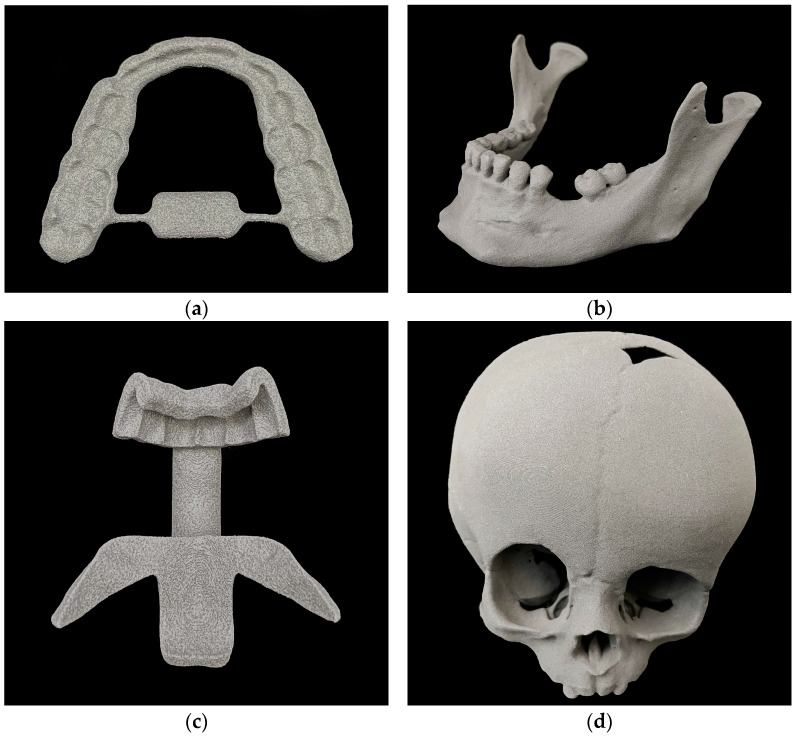
Three-dimensional prints with SLS technology: (**a**) splint, (**b**) mandibular model, (**c**) cutting guide, (**d**) skull model.

The medical applications printed with SLS technology are shown below (Figure 1).

**Figure 2:**
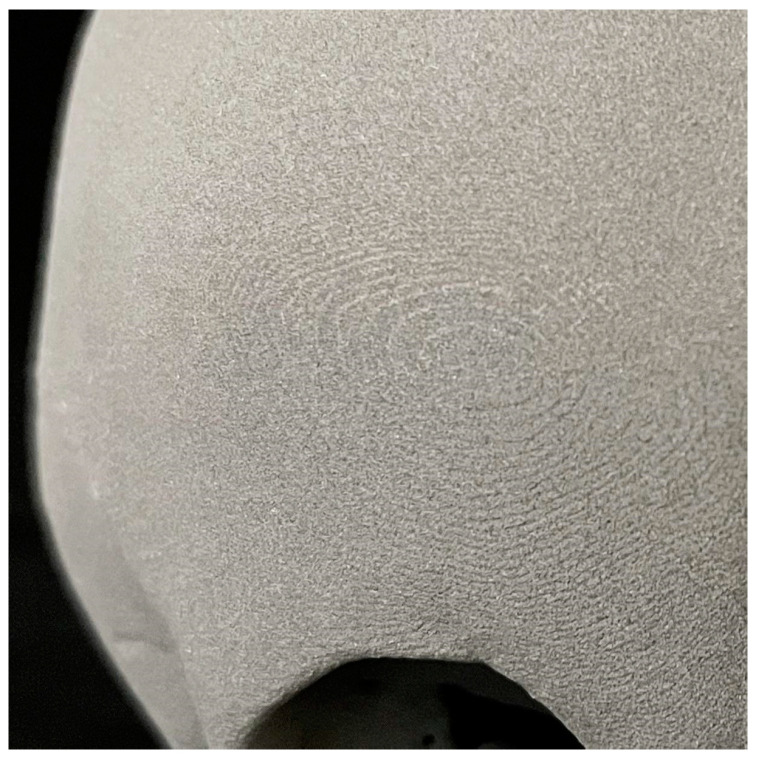
Surface of a 3D printed skull with SLS technology.

The models all had a porous and monomorphic grey surface. Occasionally, print layers were visible, especially in the skull models, as shown below (Figure 2).

**Figure 3:**
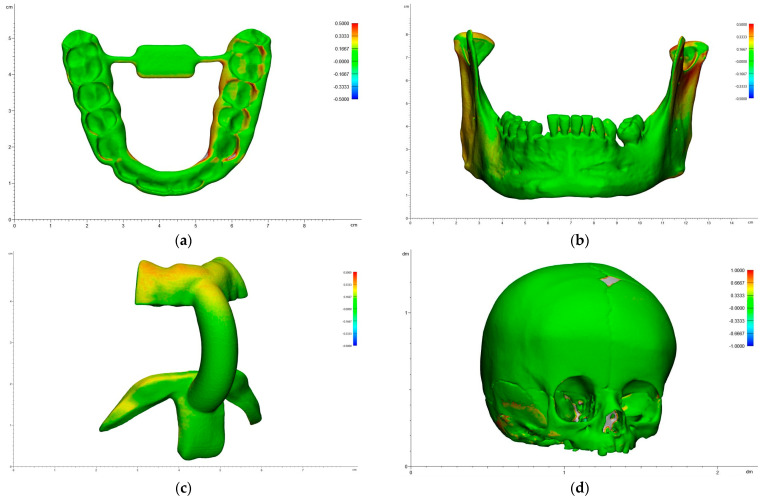
Heatmaps of the representative 3D prints with SLS technology: (**a**) splint, (**b**) mandibular model, (**c**) cutting guide, (**d**) skull model.

The heatmap shows the overlay with the deviations of a specific replica to its corresponding reference model. The red areas represent positive deviations, which indicate positions of the replica’s point cloud that are outside of the reference model. The blue deviations indicate the areas with negative deviations, which indicate positions within the replica’s point cloud that are within the reference model. Green areas signal a high level of correlation with low deviation between the replica and the reference model. The heatmaps of the representative models and applications printed with SLS technology are shown below (Figure 3).

In the splints, the highest accuracy was achieved in the incisal surface area. The lowest accuracy was achieved in the lateral molar surface region. Most of the surface was marked green and showed high accuracy (Figure 3a).

In the mandibular models, the highest accuracy was achieved in the front region. The lowest accuracy was achieved in the mandibular condyle, the coronoid process and the interdental region. Most of the surface was marked green and showed high accuracy (Figure 3b). In the cutting guides, the highest accuracy was achieved in the anterior connector arch. The lowest accuracy was achieved in the bite fixation’s external area and the chin fixation’s lateral arms (Figure 3c).

In the skull models, the highest accuracy was achieved in the nasal bone and the upper area of the parietal bone. The lowest accuracy was achieved at the lateral area of the temporal bone and the zygomatic arch. Most of the surface was marked green and showed high accuracy (Figure 3d).

#### 3.1.2. Qualitative Accuracy Assessment of SLA Printing Technology

**Figure 4:**
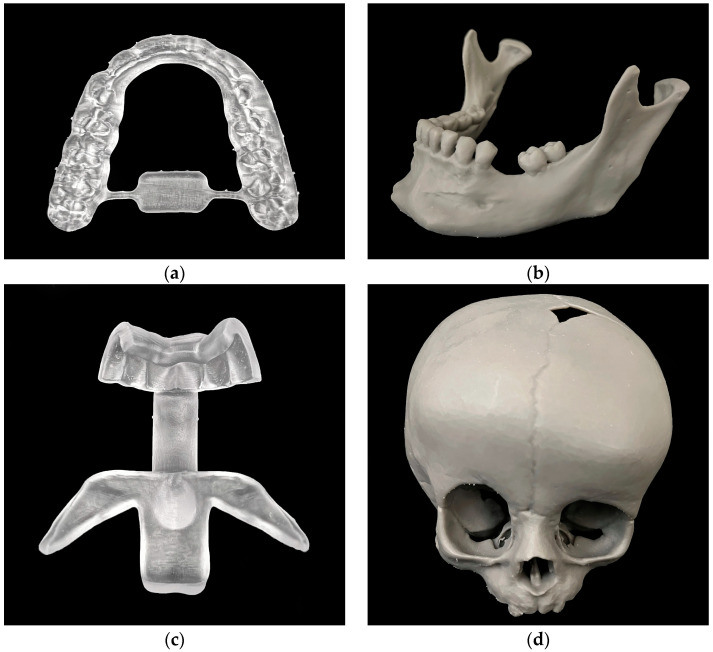
Three-dimensional prints with SLA technology: (**a**) splint; (**b**) mandibular model; (**c**) cutting guide; (**d**) skull model.

**Figure 5:**
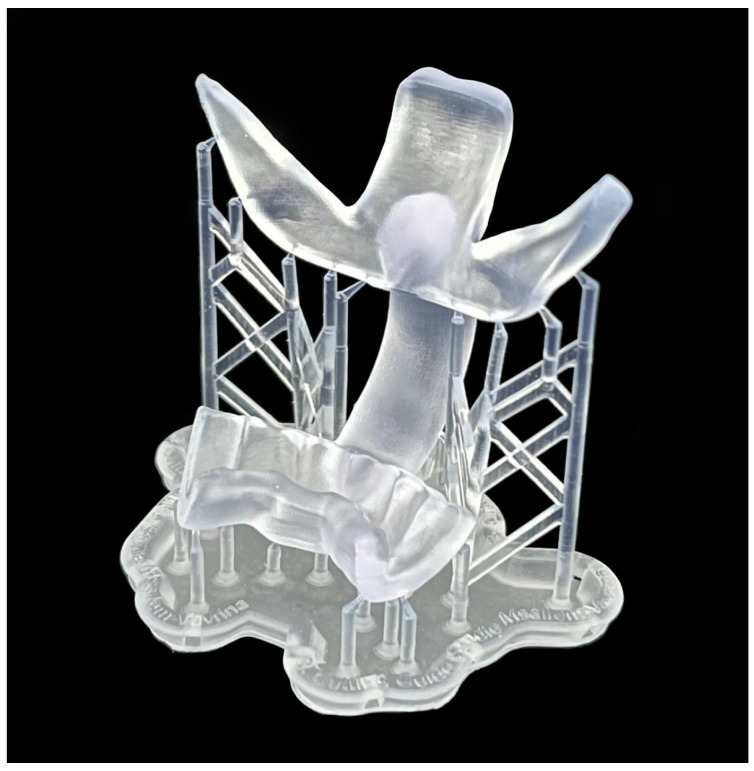
Support structure of a 3D printed cutting guide with SLA technology.

The medical applications printed using stereolithography SLA technology are shown below (Figure 4 and Figure 5). In all models, the former adhesion points of the removed supporting structures were still slightly visible.

**Figure 6:**
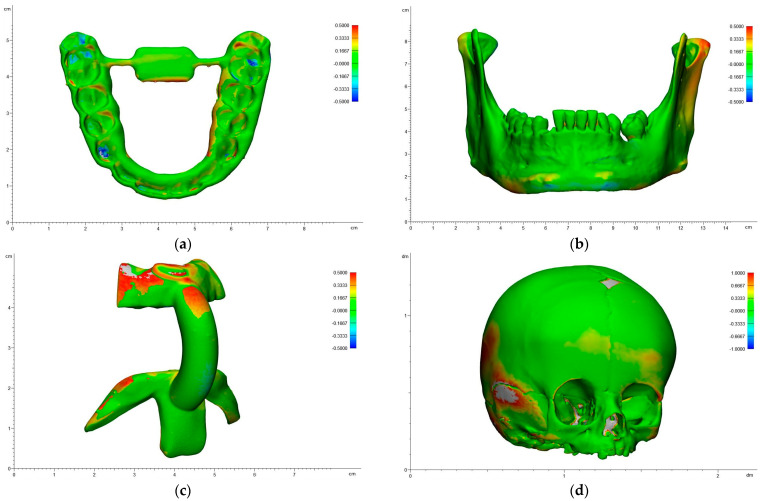
Heatmaps of the representative 3D prints with SLA technology: (**a**) splint, (**b**) mandibular model, (**c**) cutting guide, (**d**) skull model.

The heatmaps of the representative models and applications printed with stereolithography (SLA) technology are shown below (Figure 6).

In the splints, the highest accuracy was achieved in the incisal surface area. The lowest accuracy was achieved in the occlusal surface in the molar and premolar areas, where there were negative RMS values greater than 0.5 mm. Most of the surface was marked green and showed high accuracy (Figure 6a).

In the mandibular models, the highest accuracy was achieved in the front region. The lowest accuracy was achieved in the mandibular condyle, the coronoid process, the interdental, and the lower lateral mental area. In contrast to the other regions, some areas with negative RMS values were observed in the lower lateral mental area. Most of the surface was marked green and showed high accuracy (Figure 6b).

In the cutting guides, the highest accuracy was achieved in the lower anterior connector arch. The lowest accuracy was achieved in the bite fixation’s external area and the chin fixation’s lateral arms. Most of the surface was marked green and showed high accuracy, even though some deviations were greater than ±0.5 mm (Figure 6c).

In the skull models, the highest accuracy was achieved in the nasal bone and the upper area of the parietal bone. The lowest accuracy was achieved at the lateral area of the temporal bone, the zygomatic arch, the occipital region, the periorbital, and the frontal region (Figure 6d).

### 3.2. Quantitative Accuracy Assessment

#### 3.2.1. Trueness Analysis

**Figure 7:**
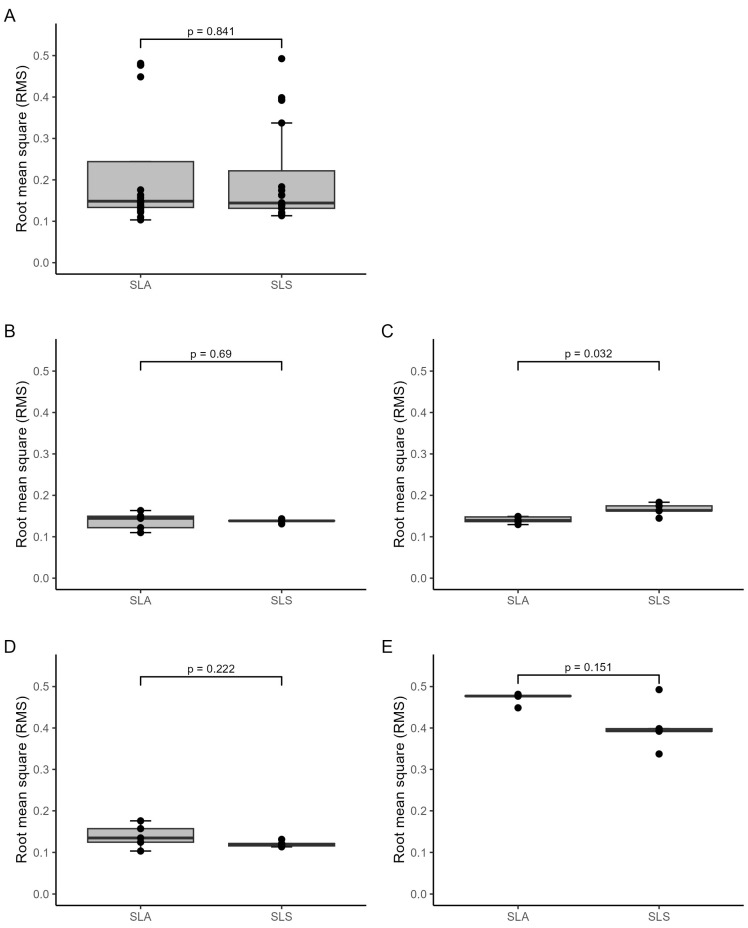
Boxplots demonstrating trueness RMS values (mm) by medical application and 3D printer type: (**A**) all models, (**B**) splints, (**C**) mandibular models, (**D**) cutting guides, (**E**) skull models.

**Figure 8:**
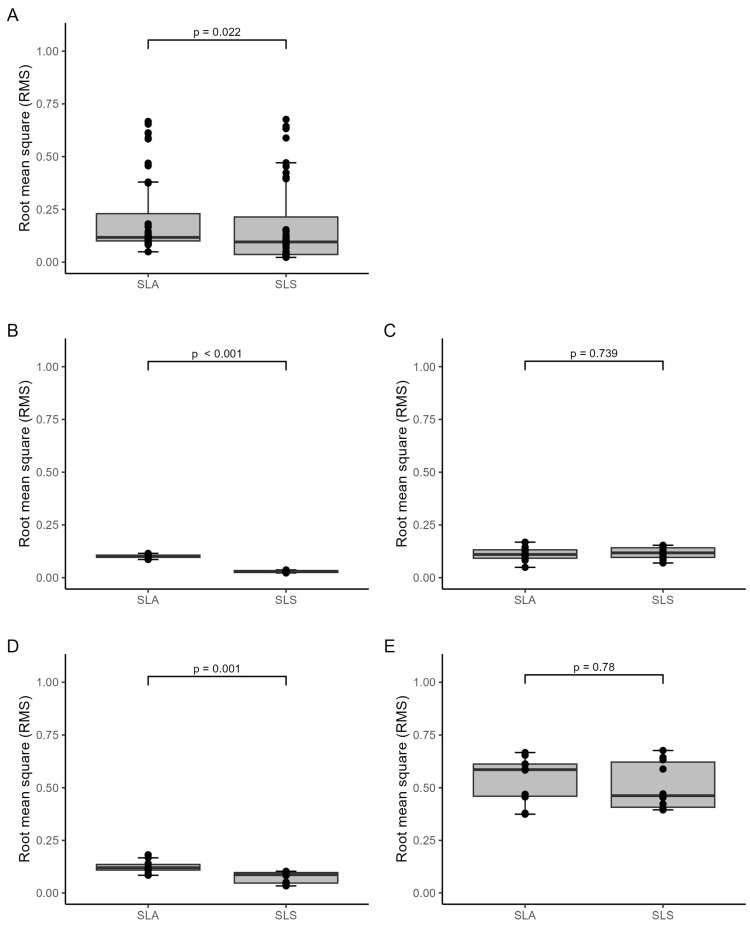
Boxplots demonstrating precision RMS values (mm) by medical application and 3D printer type: (**A**) all models, (**B**) splints, (**C**) mandibular models, (**D**) cutting guides, (**E**) skull models.

Based on the statistical analysis, no statistically significant difference was found in the overall trueness RMS values between the SLA (Form 3B+) and SLS (Fuse 1) models, combining all models. However, when examining each model type separately, a statistically significant difference was initially observed between the two printing technologies for mandibular models (Figure 7C). However, it is essential to note that this difference did not remain statistically significant after correcting for multiple tests using the Holm–Bonferroni method (*p*-adjusted) (Table 3). Upon visual inspection of the boxplots, there may be a potential difference between the two technologies for mandibular models and possibly for skull models (Figure 7E). However, it is essential to exercise caution in interpreting these visual differences because they may be purely coincidental. In some instances, SLA exhibits better trueness (mandibular models), whereas SLS performs better in other instances (skull models). Thus, these observed differences may be attributable to chance rather than a true systematic distinction. The skull models show higher RMS values than the other models as well as high maximal deviations of almost 12 mm compared to the different models and applications, with maximum deviations of 0.35–1.74 mm (Table 4). The *p*-values in Figure 7 and Figure 8 as well as Table 3 and Table 5 were determined using the Wilcoxon rank test.

#### 3.2.2. Precision Analysis

Comparing the two printing technologies, the analysis revealed a statistically significant difference: SLS technology yielded lower overall precision RMS values in the medical applications compared to the models printed with the SLA technology, considering all models together (Figure 8E and Table 5). Regarding precision specifically, RMS values were significantly lower in the splint and cutting guide applications that were printed with SLS technology than those printed with SLA technology (Figure 8B,D, as well as Table 5). In both cases, the SLS printer (Fuse 1) demonstrated more favourable precision, with lower RMS values than the SLA printer (Form 3B+). These differences remained statistically significant after conducting multiple tests, indicating robust findings. To digitise the transparent splint and cutting guide applications printed with the SLA technology, a 3D scanning spray was applied to scan the applications. All other models and applications were not transparent, and no 3D scanning spray was used. Additionally, the skull models show higher RMS values than the other models as well as high maximal deviations compared to the other models and applications, with maximum deviations of 0.18–2.54 mm (Table 6). 

**Table 5:** Comparison of the precision RMS values (mm) by 3D printer.

	Form 3B+ (SLA ^1^)		Fuse 1 (SLS ^2^)			
	*n* ^3^	Median (IQR ^4^)	*n* ^3^	Median (IQR)	*p*-Value	*p*-Adjusted *
RMS ^5^ (all replicas)	40	0.12 (0.1 to 0.23)	40	0.1 (0.04 to 0.21)	0.022	
RMS (splints)	10	0.1 (0.1 to 0.11)	10	0.03 (0.02 to 0.03)	<0.001	<0.001
RMS (mandibles)	10	0.11 (0.09 to 0.13)	10	0.12 (0.1 to 0.14)	0.739	1
RMS (cutting guides)	10	0.12 (0.11 to 0.14)	10	0.09 (0.05 to 0.1)	0.001	0.003
RMS (skulls)	10	0.59 (0.46 to 0.61)	10	0.46 (0.41 to 0.62)	0.78	1

^1^ Stereolithography, ^2^ selective laser sintering, ^3^ number of replicas, ^4^ interquartile range, ^5^ root mean square, * Holm–Bonferroni method to control for multiple testing.

**Table 6:** Summary of all precision analysis values (mm) by 3D printer.

3D Printer	Model	*n* ^1^	RMS ^2^	Mean	SD ^3^	Median	Min.	Max.
Form 3B+	all replicas	40	0.117	−0.028	0.117	−0.001	−8.982	8.12
Fuse 1	all replicas	40	0.096	−0.019	0.096	0.001	−8.536	2.971
Form 3B+	cutting guides	10	0.12	−0.007	0.119	−0.002	−1.133	1.047
Fuse 1	cutting guides	10	0.087	0.001	0.086	0.001	−1.012	0.625
Form 3B+	mandibles	10	0.11	−0.005	0.11	0	−2.544	1.083
Fuse 1	mandibles	10	0.118	0.001	0.118	0.001	−1.282	0.776
Form 3B+	skulls	10	0.586	−0.099	0.577	−0.004	−8.982	8.12
Fuse 1	skulls	10	0.462	−0.077	0.457	−0.014	−8.536	2.971
Form 3B+	splints	10	0.102	−0.001	0.102	0	−0.826	1.143
Fuse 1	splints	10	0.028	0.001	0.028	0	−0.184	0.182

^1^ Number of replicas, ^2^ root mean square, ^3^ standard deviation.

## 4. Discussion

This study tested the dimensional accuracy of various medical applications replicated using two distinct 3D printing technologies: SLA and SLS. The analysis focused on precision and trueness, which are essential parameters because inaccuracies in 3D printing within the medical field can precipitate complications and potentially harm patients. Most previous research has quantified the accuracy of anatomical models by reporting deviations in absolute millimetres rather than employing RMS values. However, RMS values more effectively account for deviations in both positive and negative directions relative to the reference model by calculating the square root of the mean squared deviations [26,38,39]. Low mean and median deviation values may lead to the erroneous conclusion that the 3D printed model is highly accurate. However, large standard deviations and large maximum and minimum deviations can lead to large RMS values corresponding to an inaccurate model. The trueness analysis, which compares the RMS values of the SLA and SLS 3D printed models to those of the reference model based on their respective Standard Tessellation Language (STL) files revealed no statistically significant differences, corroborating prior findings that both SLA and SLS technologies typically achieve high accuracy [40], with maximum deviations generally less than 1 mm, and often below 0.5 mm [9,34]. However, skull models exhibited distinctly higher RMS values, with maximal values reaching nearly 12 mm, compared to other models where deviations ranged between 0.35 and 1.74 mm. This significant discrepancy in skull models was also observed in other studies and was attributed to factors such as altered or occluded foramina, blurred sutures, and missing parts exceeding 10 mm or 1 cm^2^ in areas such as the orbit [41]. Furthermore, one study included an examination of 3D scanned and 3D printed temporal bones using an SLA 3D printer Form 2 (Formlabs Inc., Somerville, MA, USA). This study demonstrated notable deviations of up to 35% (or 2.2 mm) between the virtual model and the 3D printed outcome, and of up to 46% (or 5.01 mm) compared to the reference temporal bone. These deviations were particularly significant in smaller anatomical features such as the entrance of the carotid canal and the distance from the foramen spinosum to the anterior crest [26]. Therefore, the high deviation measured in the skull models in this study (relative to the other 3D printed models and applications) can be explained by the numerous openings, such as openings in the orbit, nasal cavity, foramen magnum, and sphenoid region. Furthermore, another study indicated that cylindrical objects with wall thicknesses of less than 3 mm were susceptible to damage during the mechanical unpacking processes [22]. This was also observed in this study, as the fragile processes at the skull base were compromised in two models, leading to deviations. Variations in scanning protocols for skull models, such as selecting a wider scan field and a larger point distance (along with increased image capture and digital processing settings) also contributed to deviations. Finally, the numerous openings in the skull models were automatically filled in digitally using the watertight setting during the meshing process. All of these factors may have led to deviations in the measurements. A study by Chae et al. confirms these findings, as mentioned above [26]. The mandibular models also exhibited minor uncaptured areas—albeit far fewer than the skull models. Areas such as the extraction alveolus require complete scanning to ensure accuracy. This is evident from the slightly increased maximum and minimum deviations in these models compared to those in the splint and cutting guide applications, which are characterised by the absence of openings or uncaptured regions. Moreover, the applications fabricated using SLA technology were affixed to support structures, necessitating their removal during postprocessing. This removal often left behind minor spikes and bumps on the surface of the models; two splints produced with SLA technology exhibited a sharp-edged overhang around the front teeth. These printing errors contributed to significant maximum deviations but had a minimal impact on the RMS values due to their localised nature.

A principal source of inaccuracy was likely the application of a white 3D scanning spray on the splints and cutting guides produced using SLA technology. The appropriate resin was used for each medical application. Therefore, unlike the other models, the cutting guides and splints (printed with SLA technology) were created out of a transparent biocompatible resin, BioMed Clear (Formlabs Inc., Somerville, MA, USA), necessitating the use of Reflecon Tarnish 11 3D scanning spray (MR Chemie GmbH, Unna, Germany) to facilitate (optical) structured-light scanning. This treatment resulted in notably higher RMS values in the precision analysis of these applications compared to those printed with SLS technology using the Fuse 1 printer. Notably, the lowest accuracy was observed on the occlusal surfaces in the molar and premolar areas of the SLA-printed splints, where negative RMS values were recorded. This inaccuracy may have been caused by irregularities in the application of the 3D scanning spray, where insufficient coverage led to deeper light penetration and reflection inconsistencies during the scanning process. The reproducibility of this study protocol was confirmed by the consistency of inaccuracies in the mandibular models, which mirror those reported in the reference study by Msallem et al. [9]. The highest accuracy was achieved in more robust regions such as the front region, mandibular corpus, and mandibular angle. Conversely, the lowest accuracy was observed in peripheral parts such as the mandibular condyle and coronoid process, or in regions with immersions such as interdental spaces and the extraction alveolus. Other studies corroborate the finding that centrally located model parts are typically printed with higher accuracy, whereas peripheral parts (or those with immersions) are less accurately printed [22]. Furthermore, thinner areas displayed less stability and were prone to deformation during the curing process [22,42].

Previous studies by the authors have identified workflows to facilitate facial reconstruction, considering feasibility, cost-effectiveness, force resistance and accuracy, with the choice of a particular 3D printer [9,38,43,44]. In one of those studies, the SLS 3D printer EOSINT P 385 (EOS GmbH, Krailling, Germany) performed best out of all 5 printers tested, and the SLA 3D printer Form 2 (Formlabs Inc., Somerville, MA, USA) performed worst [9]. Table 7 and Table 8 below show the results of the 3D printers used in this study compared to those used in the comparative study. It should be noted that the original mandibular STL file was the same, whereas the structured light scanner was different in both studies.

Comparing SLA technology, the Form 3B+ shows higher accuracy in terms of trueness relative to the Form 2. Comparing SLS technology, the EOSINT P 385 shows slightly higher accuracy in terms of trueness relative to the Fuse 1.

Analysis of SLA technology revealed that the Form 3B+ offers comparable precision in RMS values to that of the Form 2, albeit with a significantly reduced SD, indicating improved overall performance (likely due to advancements in the newer model). In the realm of SLS technology, the EOSINT P 385 mirrored the precision of the Fuse 1, although the RMS value was marginally lower, and variability in minimum and maximum deviations was slightly higher. The EOSINT P 385 remains slightly more accurate than the Fuse 1. It is also noteworthy that the EOSINT P 385 is the most expensive of the printers evaluated.

The planning phase is vital in 3D printing. The print direction in space significantly impacts the accuracy and structural integrity of the models through the orientation and the number of layers required [29,30,45]. In contrast to SLS printing, SLA printing requires support structures for larger objects; furthermore, their number, insertion, and orientation also significantly impact the stability and accuracy of the object [32,33]. In the referenced and current studies, models were vertically aligned, preserving the occlusal surface and minimising the need for support structures [9]. Layer thickness also significantly impacts the accuracy of 3D printed models [28]. The layer thickness of the SLA printer used in this study was 100 µm, but it would also be possible to print layer thicknesses of up to 25 µm with the Form 3B+. Yet studies have shown that layer thicknesses below 100 µm do not necessarily achieve greater accuracy [31,46]. However, given that the SLS printer can print layer thicknesses only up to 110 µm, layer thicknesses were aligned to improve comparability between the two printers. Compared with other studies investigating the accuracy of the Fuse 1 [23,47] or the Form 3B+ [48,49,50,51], similar values were found for mean, median and maximum deviations; RMS values were comparable as well.

Despite such insights, this study is limited by the modest sample size of five models per printer type and technology. This may have underpowered the detection of statistically significant differences. Nonetheless, consistent deviation values observed in an STL file of the mandible used in the previous and current studies suggest that the findings are representative. Factors such as scanner vibrations and minor fluctuations from the scan table were anticipated, and this study paid close attention to detail in measurement practises. Even the slight layer of scanning spray applied during data acquisition was noted to affect the measurements.

As 3D printing technology evolves, printers continue to vary widely in design, functionality and material compatibility, albeit with generally high accuracy among contemporary models. However, the diversity in material processing capabilities and print speed remains a bottleneck in broader applications within the field. These results are also reflected by the current development of 3D printers. This study’s findings indicate that future improvements will focus primarily on enhancing the speed of prints and the size of printed objects (with less emphasis on accuracy because high levels of accuracy can already be achieved). As part of its ongoing technical advancement, Formlabs has recently unveiled the successor models to the Form 3B+ and the successor to the Fuse 1: the Form 4B and the Fuse 1+, respectively. The following advancements are in accordance with the above predictions. The main distinction between Form 3B+ and Form 4B is the speed and the exposure unit. The Form 3B+ employs a single laser unit, whereas the Form 4B utilises 60 LEDs. This technology is called masked stereolithography instead of laser-based stereolithography. Objects with a production volume of up to 5.25 L can be produced with the Form 4B, whereas objects of up to 4.05 L can be produced with the Form 3B+. The number of materials to be processed and the desired layer thickness are identical across both models. However, the Form 4B comes at a significantly higher price. The principal distinction between the Fuse 1 and the Fuse 1+ lies in the production speed and the number of materials that can be processed. Additionally, the printing environment of Fuse 1+ comprises inert gas and air, whereas Fuse 1 employs pure air. The maximum component size and layer thickness remain identical. However, the price of the Fuse 1+ is also higher than that of its predecessor model.

Following recommendations from an earlier study, the authors confirmed that the printers studied here consistently deliver similar accuracy across different model sizes for each technology type. An ongoing objective remains to establish standardised testing methods for 3D printed models using advanced digital measurement techniques, particularly for 3D printing in healthcare applications.

## 5. Conclusions

In this study, the SLS printer (Fuse 1) demonstrated the highest overall accuracy with precision and trueness (RMS: 0.096 and 0.144 mm), whereas the SLA printer (Form 3B+) exhibited slightly lower values (RMS: 0.117 and 0.148 mm). Considering that most deviations are in the micrometre range, it is highly unlikely that these minor deviations in medical applications will affect surgical outcomes. Overall, both 3D printers showed high accuracy, and the values measured here are consistent with those reported in the literature. Therefore, both printers using different printing technologies (i.e., SLA and SLS) are good choices for patient-specific treatment. However, SLA technology has a more extensive variety of printing materials currently available. In conclusion, our study underlines the need for a paradigm shift in selecting 3D printers for medical applications. Traditionally, the choice of printer technology has dominated the decision-making process; today, however, the intended medical application and compatibility with different printing materials relative to budget constraints are more critical considerations. Entry-level SLS printers have gained importance in recent years, especially for point-of-care 3D printing, as they have dropped significantly in purchase price. The need to use and validate certified, biocompatible materials still limits the scope of this technology. The affordability and material versatility of SLA printers increase their value and make them a compelling complement to Fused filament fabrication (FFF) printers, which are used in many educational and point-of-care scenarios. Of note is the ability of SLA printers to process biocompatible, sterilisable materials, which are essential for producing high-precision surgical guides such as drilling and cutting guides. This capability currently represents a significant advantage over FFF printers in the medical field. In addition, the development of 3D printing in medical facilities is rapidly moving from the creation of mere anatomical models and pre-bending tools to the production of surgical guides and splints—and potentially to customised implants tailored to the patient’s individual needs and anatomy. However, the trajectory of this development depends not only on technological advances and cost efficiency but also on strict regulatory frameworks for medical device manufacturing. As the landscape of medical 3D printing continues to evolve, it is imperative that healthcare providers and medical device manufacturers consider these factors in their strategic planning and adoption of the technology. This approach will ensure that the benefits of 3D printing technology can be fully realised in clinical settings, thereby enhancing patient care and surgical outcomes.

## Figures and Tables

**Table 1:** Three-dimensional (3D) printer specifications and material.

3D Printer	Manufacturer	Technology	Layer Thickness	Material
Fuse 1	Formlabs Inc., Somerville, MA, USA	SLS ^1^	110 microns	PA12 (polyamide powder)
Form 3B+	Formlabs Inc., Somerville, MA, USA	SLA ^2^	100 microns	Standard Grey (resin)/BioMed Clear (resin)

^1^ Selective Laser Sintering, ^2^ Stereolithography.

**Table 2:** Three-dimensional (3D) scanner specifications.

Device	Manufacturer	Point Distance	Scan Mode
Transcan C	Shining 3D Tech. Co., Ltd., Hangzhou, China	0.0375 mm/0.075 mm	Structured light

**Table 3:** Comparison of the trueness RMS values (mm) by 3D printer.

	Form 3B+ (SLA ^1^)		Fuse 1 (SLS ^2^)			
Model	*n* ^3^	Median (IQR ^4^)	*n* ^3^	Median (IQR)	*p*-Value	*p*-Adjusted *
RMS ^5^ (all replicas)	20	0.15 (0.13 to 0.24)	20	0.14 (0.13 to 0.22)	0.841	
RMS (splints)	5	0.14 (0.12 to 0.15)	5	0.14 (0.14 to 0.14)	0.69	0.69
RMS (mandibles)	5	0.14 (0.14 to 0.15)	5	0.16 (0.16 to 0.17)	0.032	0.128
RMS (cutting guides)	5	0.13 (0.12 to 0.16)	5	0.12 (0.12 to 0.12)	0.222	0.453
RMS (skulls)	5	0.48 (0.48 to 0.48)	5	0.39 (0.39 to 0.4)	0.151	0.453

^1^ Stereolithography, ^2^ selective laser sintering, ^3^ number of replicas, ^4^ interquartile range, ^5^ root mean square, * Holm–Bonferroni method to control for multiple testing.

**Table 4:** Summary of all trueness analysis values (mm) by 3D printer.

3D Printer	Model	*n* ^1^	RMS ^2^	Mean	SD ^3^	Median	Min.	Max.
Form 3B+	all replicas	20	0.148	0.093	0.133	0.048	−1.181	11.81
Fuse 1	all replicas	20	0.144	−0.038	0.116	−0.06	−0.813	11.969
Form 3B+	cutting guides	5	0.135	0.034	0.135	0.022	−0.944	0.909
Fuse 1	cutting guides	5	0.12	−0.07	0.096	−0.057	−0.349	0.919
Form 3B+	mandibles	5	0.14	0.043	0.132	0.027	−0.6	1.742
Fuse 1	mandibles	5	0.164	−0.073	0.144	−0.066	−0.813	1.646
Form 3B+	skulls	5	0.477	0.214	0.429	0.118	−1.181	11.81
Fuse 1	skulls	5	0.393	0.081	0.39	−0.011	−0.73	11.969
Form 3B+	splints	5	0.144	0.078	0.114	0.058	−0.669	1.192
Fuse 1	splints	5	0.138	−0.093	0.102	−0.09	−0.592	0.351

^1^ Number of replicas, ^2^ root mean square, ^3^ standard deviation.

**Table 7:** Trueness analysis values (mm) for the 3D printers included in the present study and in the comparative study.

3D Printer	Technology	Model	RMS ^1^	Mean	SD ^2^	Median	Min.	Max.
Form 3B+	SLA	mandibles	0.14	0.043	0.132	0.027	−0.6	1.742
Form 2 *	SLA	mandibles	0.45	0.23	0.39	0.17	−1.91	1.69
Fuse 1	SLS	mandibles	0.164	−0.073	0.144	−0.066	−0.813	1.646
EOSINT P 385 *	SLS	mandibles	0.11	−0.07	0.08	−0.06	−0.51	0.87

^1^ Root mean square, ^2^ standard deviation, * comparative study by Msallem et al. [9].

**Table 8:** Precision analysis values (mm) for the 3D printers included in the present study and in the comparative study.

3D Printer	Technology	Model	RMS ^1^	Mean	SD ^2^	Median	Min.	Max.
Form 3B+	SLA	mandibles	0.11	−0.005	0.11	0	−2.544	1.083
Form 2 *	SLA	mandibles	0.09	0.01	0.24	0	−1.73	1.67
Fuse 1	SLS	mandibles	0.118	0.001	0.118	0.001	−1.282	0.776
EOSINT P 385 *	SLS	mandibles	0.07	0	0.17	0	−1.30	1.18

^1^ Root mean square, ^2^ standard deviation, * comparative study by Msallem et al. [9].

## Data Availability

Data are contained within the article.

## Abbreviations

3D	Three-dimensional
AM	Additive manufacturing
CAD	Computer-aided design
CAM	Computer-aided manufacturing
CT	Computed tomography
DICOM	Digital imaging and communications in medicine
FFF	Fused filament fabrication
IQR	Interquartile range
MB	Megabyte
PA12	Polyamide powder
RMS	Root mean square
SD	Standard deviation
SLA	Stereolithography
SLS	Selective laser sintering
STL	Standard Tessellation Language
